# SAA1 Expression as a Potential Prognostic Marker of the Tumor Microenvironment in Glioblastoma

**DOI:** 10.3389/fneur.2022.905561

**Published:** 2022-06-10

**Authors:** Kangxi Cao, Xingyu Jiang, Baishun Wang, Zhaohui Ni, Yan Chen

**Affiliations:** ^1^Department of Neurosurgery, The Second Hospital of Jilin University, Changchun, China; ^2^Department of Pathogenobiology, The Key Laboratory of Zoonosis, Chinese Ministry of Education, College of Basic Medical Sciences, Jilin University, Changchun, China

**Keywords:** SAA1, glioblastoma, immune infiltration, tumor microenvironment, prognosis

## Abstract

**Background:**

Glioblastoma (GBM) is the most common primary brain malignant tumor, and patients with GBM have a poor prognosis. The tumor microenvironment (TME) is connected to tumorigenesis and prognosis. However, the TME-related genes and therapeutic targets in GBM are yet to be identified. Thus, the presented study aimed to identify TME-related biomarkers in GBM and develop a novel target for the treatment of the disease.

**Methods:**

ESTIMATE computational methods were utilized to estimate the amounts of stromal and immune components in 697 patients with glioma from the Cancer Genome Atlas database. Then, the protein–protein interaction network and univariate Cox regression analyzed the differentially expressed genes. Serum amyloid A1 (SAA1) was determined to be a predictive factor. SAA1 expression was statistically significant in GBM compared to the normal samples and other glioma subtypes and negatively associated with survival. Independent prognostic analysis identified SAA1 as a TME-related prognostic factor. Furthermore, Western blot analysis showed that SAA1 is upregulated in GBM, which was confirmed by the external validation in the Chinese Glioma Genome Atlas. The gene set enrichment analysis in GBM revealed enrichment of immune-related activities in the SAA1 high-expression group, while mitosis and cell cycle were enriched in the low-expression group. CIBERSORT analysis of the tumor-infiltrating immune cell proportion revealed that M2 macrophages, neutrophils, activated mast cells, resting mast cells, and regulatory T cells were correlated with SAA1 expression. Finally, immune checkpoint genes, tumor mutation burden, and drug sensitivity were also analyzed between the high- and low-expression groups.

**Conclusion:**

SAA1 could be a distinctive gene between GBM and other subtype gliomas, and thus a novel biomarker for estimating the survival and TME status. The altered expression level shifts the primary function of SAA1 from cell cycle and mitosis to immune activity. High expression of SAA1 is associated with poor survival and upregulates the expression of LAIR1 and TNFSF14, thereby deeming it as the drug sensitivity indicator for XAV939, TGX-221, and lapatinib in GBM immune therapy.

## Introduction

Gliomas are the most common primary brain tumors, accounting for about 80% of central nervous system malignancies (1). These tumors are divided into four grades according to the World Health Organization classification. Low-grade gliomas (LGGs) generally refer to grade 2 gliomas, whereas grades 3 and 4 are high-grade gliomas (HGGs) (2). LGGs are often used to refer to grades 2 and 3 gliomas, consistent with the Cancer Genome Atlas (TCGA) database (1). HGGs have a poor prognosis. The median overall survival (OS) of grade 3 gliomas is 3 years, while that of grade 4 gliomas, especially glioblastoma (GBM), is about 15 months, with only 0.05–4.7% of patients surviving for 5 years after diagnosis (3, 4).

The treatment of primary gliomas comprises surgical resection, radiotherapy, and chemotherapy (1). Although glioma patients could benefit from these treatment strategies, the OS time is still short. Thus, in order to enhance the survival rate of glioma patients, it is critical to identify a precise and effective prognostic factor and a therapeutic target.

The occurrence and development of gliomas are complex. Gene mutations, chromosomal abnormalities, and changes in the cellular environment can lead to tumorigenesis. Gliomas are masses of malignant cells that damage the functional health of other cells in the body (5). The imbalance between healthy and malignant cells influences the prognosis and leads to unsatisfactory outcomes, including death (5, 6). The tumor microenvironment (TME) is a complex heterogeneous system consisting of tumor cells, stromal cells, and immune cells. Currently, increasing evidence indicates that TME is linked to cancer cell proliferation, invasion, and metastasis (7, 8). Previous studies have shown that in the TME, immune activation and escape could occur before cancer invasion (9). Some recent studies have revealed that the TME in the brain is a critical regulator of cancer progression and therapeutic efficacy (9). The subtypes and functions of tumor-infiltrating immune cells (TICs) in the TME of glioblastoma (GBM) have also been explored (10). However, the biomarkers associated with glioma TME and their value in prognosis and treatment have not been reported. Therefore, we aimed to explore the transformation and associated genes in the TME of GBM.

In this work, based on the transcriptome RNA-seq data and clinical characteristics of glioma samples from the TCGA database, integrated bioinformatics methods were used to calculate the TME scores, estimate the number of TICs, and screen differentially expressed genes (DEGs) related to immune components. SAA1 was identified as a candidate TME-related hub gene with potential functions associated with prognosis for GBM patients. Thereafter, expressions of SAA1 were successfully verified in an external database Chinese Glioma Genome Atlas (CGGA) and glioma cell lines (U87MG, and U251). The gene set enrichment analysis in GBM revealed enrichment of immune-related activities in the SAA1 high-expression group. CIBERSORT analysis revealed that M2 macrophages, neutrophils, activated mast cells, resting mast cells, and regulatory T cells were correlated with SAA1 expression. Immune checkpoint genes, tumor mutation burden, and drug sensitivity were also analyzed between the high- and low-expression groups. All the results demonstrated that SAA1 might be a new TME-related biomarker of GBM and has the potential for prognosis and therapeutic target.

## Materials and Methods

### Bioinformatics Database

**Figure 1:**
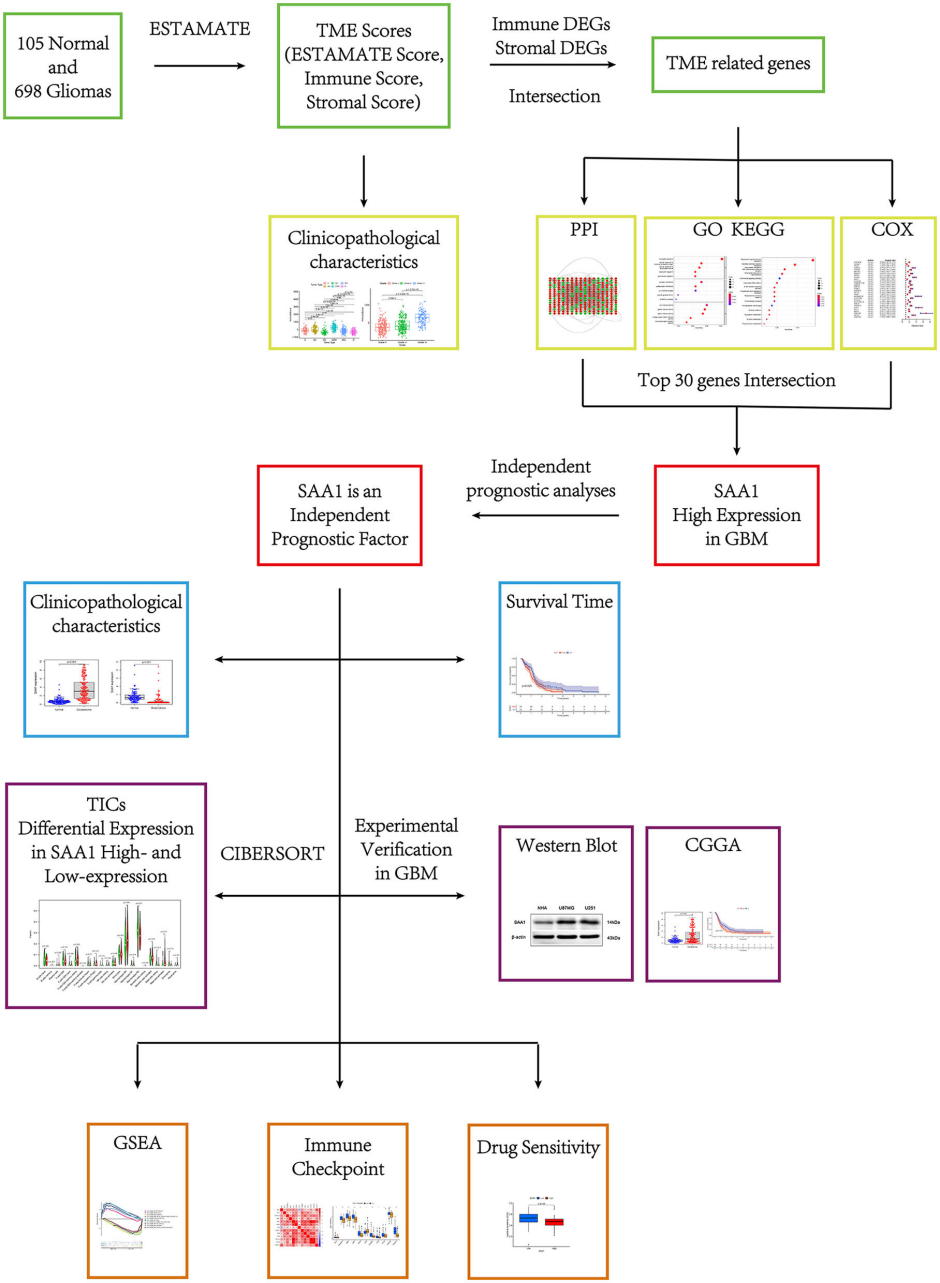
Research process of this study.

The flow of this study is shown in Figure 1. The transcriptome RNA-seq data of 105 normal cases and 697 glioma cases (168 GBM and 529 LGGs) and the corresponding clinical data were downloaded from the UCSC Xena database (http://xena.ucsc.edu/). Normal samples were obtained from the GTEX dataset, and glioma samples were from GDC TCGA LGG and GDC TCGA GBM datasets. The samples without complete clinical information and overall survival (OS) <30 days were moved. Finally, 105 normal, 158 GBM, and 493 LGG samples were utilized for subsequent analyses. For external validation, the dataset, including RNA-seq and clinical data, was obtained from the CGGA database.

### Generation of TME Scores

The ESTIMATE algorithm run in R language (version 4.0.2) was used to estimate the proportion of immune and stromal components in the TME of each sample and generate three scores: immune, stromal, and ESTIMATE. These were positively correlated with the proportions of immune, stromal, and the sum of both components, respectively. Thus, the higher the score, the higher the proportion of the relevant component in the TME.

### TME Scores Correlated Analyses

We used the R packages, survival, survminer, lima, and ggpubr to estimate the TME scores and conduct the related analyses. To reduce statistical bias in this analysis, we excluded glioma patients with missing and short OS values (<30 days). The Kaplan–Meier (KM) method was used to plot the survival curve, and the log-rank test was used to determine the statistical significance; *p* < 0.05 was considered significant. Furthermore, the difference in TME scores in clinical characteristics, glioma grade, and glioma subtype was shown in the plots.

### Identification of the TME-Related Genes Based on Immune and Stromal Scores

Glioma samples were assigned a high or low score depending on the difference in the median values for the immune and stromal scores. The R package limma was used for the differential analysis of gene expression, and differentially expressed genes (DEGs) were identified by comparing the high-score samples to the low-score samples. DEGs with fold-change (FC) > 1 after transformation of log2 (high-score group/low-score group) and a false discovery rate (FDR) <0.05 were considered significant. Then, the genes that overlapped in the intersection of the immune and stromal DEGs were considered TME-related genes. The subsequent analyses were based on the TME-related genes.

### Gene Ontology (GO) and Kyoto Encyclopedia of Genes and Genomes (KEGG) Enrichment Analyses

Gene ontology and KEGG enrichment analyses of the TME-related genes were performed using R language with the clusterProfiler, enrichplot, and ggplot2 packages. Only terms with *p* < 0.05 were considered significantly enriched.

### Identification of SAA1 as a Prognostic Gene in GBM

A protein-protein interaction (PPI) network was constructed for the TME-related genes using the STRING database (https://cn.string-db.org/), followed by reconstruction using the Cytoscape software (version 3.7.2). Nodes with the confidence of interactive relationships > 0.4 were used for constructing the network. Then, the degrees (the number of associated lines from a gene to other genes) of every node were calculated.

R package, survival, was used for univariate Cox (uni-Cox) regression analysis and KM survival; *p* < 0.05 indicated statistical significance. The top 30 genes were identified by increasing *p*-values in uni-Cox analysis.

According to the PPI and uni-Cox results, SAA1 was the only prognostic gene identified from the top 30 genes acquired from the PPI and uni-Cox analyses. Furthermore, R packages, limma, survminer, and beeswarm were used to show the expression and survival status of SAA1 between normal and differential characteristic gliomas in order to further explore the association between *SAA1* and glioma.

### Identification of SAA1 as an Independent Factor for Bioinformatic Analyses

To evaluate the risk score and clinical characteristics, uni-Cox and multivariate Cox (multi-Cox) regression were applied, and receiver operating characteristic (ROC) curves were plotted to compare the different factors and predict the outcomes. Then, the R package rms utilized SAA1 expression, age, and gender to set up a nomogram for the 1-, 3-, and 5-year OS. Furthermore, the correction curves based on the Hosmer–Lemeshow test illustrated whether the prediction outcome was consistent with the practical results.

### Cell Culture

NHA, U87MG, and U251 cell lines were grown under 5% CO_2_ at 37°C in Dulbecco's modified eagle medium (DMEM; Gibco, NY, USA) supplemented with 10% fetal bovine serum and 1% penicillin-streptomycin. All cell lines in this study were obtained from the Shanghai Cell Bank of the Chinese Academy of Medical Sciences (Shanghai, China).

### WB Analysis

Cells were lysed with radioimmunoprecipitation assay (RIPA) lysis buffer (Beyotime, China) containing 1% phenylmethanesulfonyl fluoride (PMSF; Beyotime). The protein concentration was measured using a BCA protein assay kit (Beyotime, China). Equal amounts of proteins were separated by 12% sodium dodecyl sulfate poly acrylamide gel electrophoresis (SDS-PAGE), transferred to polyvinylidene fluoride membranes, and probed with SAA1 and β-actin antibodies (Bioss, Beijing, China) at 4°C overnight. Subsequently, the membranes were incubated with horseradish peroxidase (HRP) Affini Pure goat anti-rabbit IgG secondary antibody at room temperature for 2 h. The immunoreactive bands were detected with appropriate Electro chemiluminescence chromogenic substrates on a Chemiluminescence Imaging System (GeneGnome XRQ, UK).

### External Validation in the CGGA Database

The GBM mRNA sequencing and clinical data were downloaded from the CGGA database (http://cgga.org.cn/index.jsp), and the GBM patient and associated clinical characteristics were extracted. Furthermore, the differences in SAA1 expression and survival status were compared between *SAA1* high- and low-expression groups; *p* < 0.05 indicated statistical significance.

### Gene Set Enrichment Analysis (GSEA)

A tripartite GSEA analysis was conducted. Hallmark collections were downloaded from the Molecular Signatures Database (11), GO, KEGG collections, and GSEA 4.1.0 software were downloaded from Broad Institute. The SAA1 expression level of GBM patients was classified into low- and high-group. In addition, the transcriptome data were utilized for GSEA, and only gene sets with nominal (NOM) *p* < 0.05 were considered significant.

### TIC Profile

The CIBERSORT computational method was applied to estimate the TIC abundance in GBM samples, followed by quality-filtering. Then, the difference in immune cells between the SAA1 low- and high-expression groups were compared, and the results acquired were deemed SAA1-related TICs.

### SAA1-Associated Immune Checkpoint Analysis and TMB

Immune checkpoint-related genes were obtained from previous studies, and GBM patients were grouped into low- and high-expression groups based on the median of SAA1 expression. Pearson's test was applied to assess the correlation between SAA1 and immune checkpoint genes, and the correlation coefficient >0.4 was considered significant. The expression of immune checkpoint genes was compared between the two groups, and *p* < 0.05 was considered statistically significant. The overlapping genes between the Pearson's and differential expression tests were SAA1-associated immune checkpoint genes. The tumor mutation data were downloaded from the TCGA database (https://portal.gdc.cancer.gov/). Finally, the mutation of SAA1 and associated immune checkpoint genes were estimated.

### Exploration of SAA1 Expression Level in the Clinical Treatment

R package pRRophetic was employed to evaluate SAA1 therapy response, as determined by the half-maximal inhibitory concentration (IC50) of each GBM patient in Genomics of Drug Sensitivity in Cancer (GDSC) (https://www.cancerrxgene.org/) (12).

### Statistical Analyses

R 4.0.2 software was used to analyze the data. For survival analysis, the samples were divided into high- and low-expression groups based on the median value and statistically analyzed by the KM method. For gene expression difference analysis and drug sensitivity, samples were divided into high- and low-expression groups based on the median value method and screened by Wilcoxon rank-sun test. For survival-related genes, the KM method and uni-Cox analysis were used for screening, and Pearson's correlation coefficient was used for screening SAA1-related immune checkpoint genes. The protein levels of WB are presented as the mean ± standard error of the mean (SEM) and screened by *t*-test. In the above statistical tests, *p* < 0.05 or FDR < 0.05 was considered statistically significant.

## Results

### TME Scores Were Associated With the Survival and Clinical Characteristics of Glioma Patients

**Figure 2:**
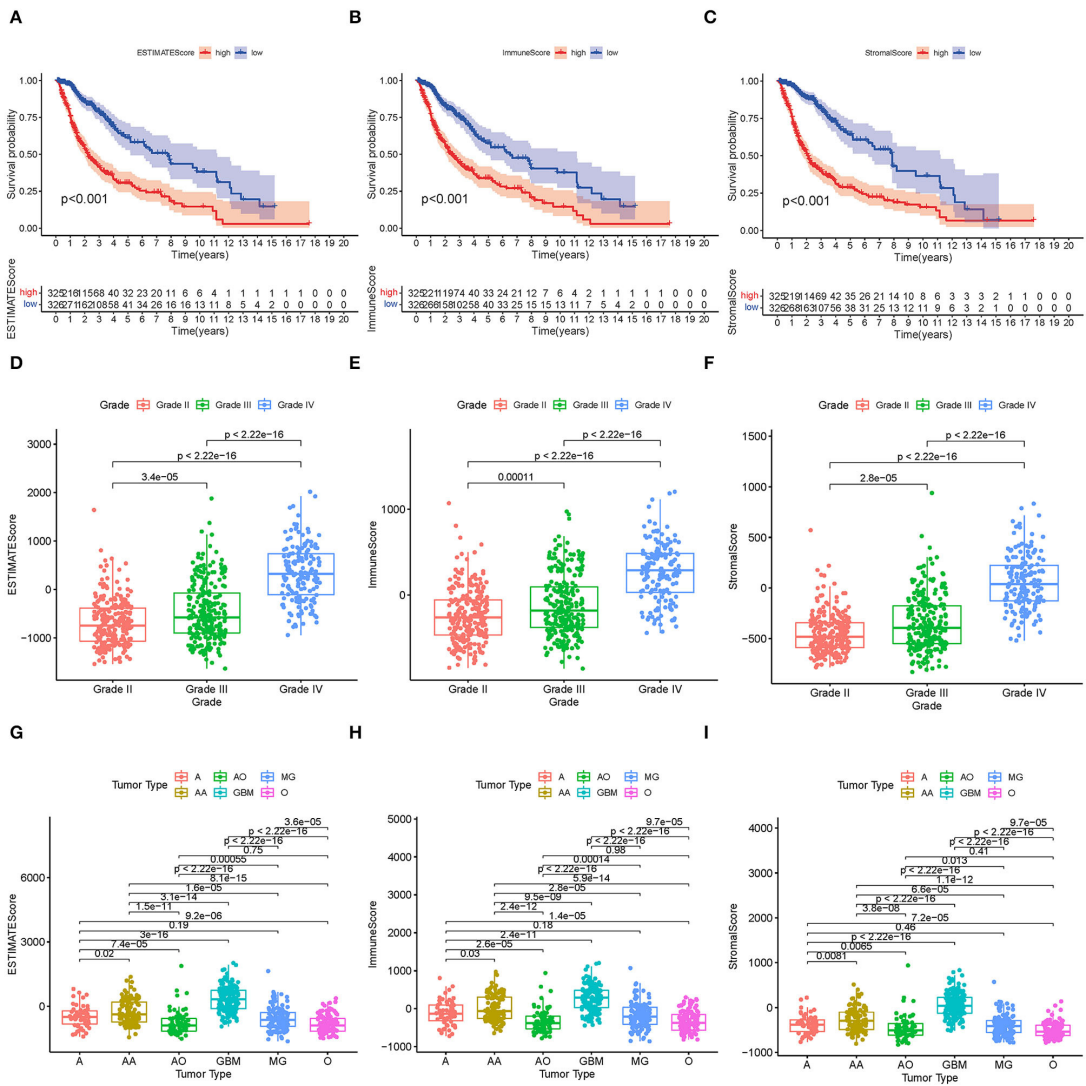
Correlation between TME scores and survival and clinical characteristics. **(A–C)** KM survival analysis for glioma patients, grouped by low- and high-TME scores compared to the median. **(D–F)** Distribution of the ESTIMATE score, immune score, and stromal score by glioma grade. **(G–I)** Distribution of the ESTIMATE score, immune score, and stromal score by glioma type.

The KM survival analysis was used to analyze the immune, stromal, and ESTIMATE scores to establish the association of the estimated proportions of immune and stromal cells with the survival time. A high immune or stromal score indicated high amounts of the immune or stromal components in the TME. The ESTIMATE score is the sum of the immune and stromal scores, indicating the proportions of both immune and stromal components in the TME. Figures 2A–C shows that the immune, stromal, and ESTIMATE scores are negatively correlated with the OS. Although this negative correlation indicates that both immune and stromal components influence the prognosis of glioma, we selected the immune component to predict the prognosis in subsequent analysis. In order to determine the internal correlation between the proportion of immune and stromal components with the clinicopathological features, we analyzed the corresponding clinical information of the glioma cases. As shown in Figures 2D–I, the immune, stromal, and ESTIMATE scores showed a positive correlation with the glioma grade and type, especially for GBM. Also, grade 4 glioma was represented, indicating positive scores compared to other grade and subtype gliomas (all *p* < 0.001). These results suggested that the proportion of the immune and stromal components was associated with glioma progression concerning invasion and survival time, especially in grade 4 glioma and GBM.

### TME-Related Genes Based on the Immune and Stromal Scores Were Presented as an Enrichment of Immune-Related Genes

**Figure 3:**
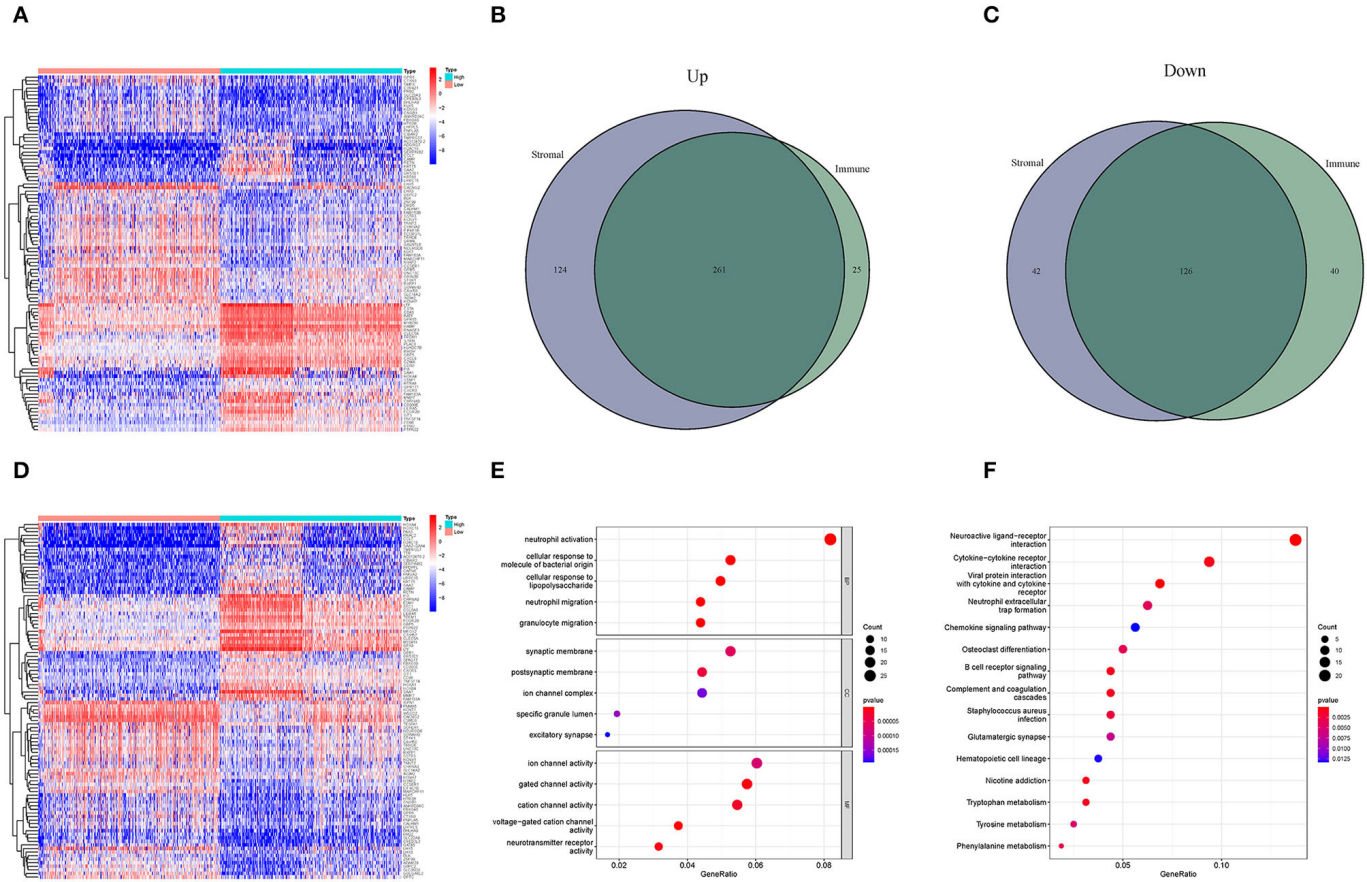
Heatmaps and the interaction of DEGs, GO, and KEGG analyses. **(A,D)** Heatmaps for DEGs were generated by comparing the high-score group vs. the low-score group. The row name is the gene, and the column name is the sample ID, not shown in the image. DEGs were identified by Wilcoxon rank-sum test with FDR < 0.05 and | log_2_FC (fold-change) | > 1. **(A,D)** Represent the immune and stromal scores, respectively. In the two heatmaps, only 50 DEGs are shown. **(B)** Interaction of upregulated DEGs with immune score and stromal score. **(C)** Interaction of downregulated DEGs with immune score and stromal score. **(E,F)** GO and KEGG enrichment analyses for 452 DEGs; *p* < 0.05 were speculated to be enriched significantly.

High- and low-score glioma samples were compared to ascertain the exact changes in gene profiles in the TME for the immune and stromal components. A total of 452 DEGs were identified based on the immune score (comparing samples with high and low scores): 286 were upregulated and 166 were downregulated (Figures 3A–C). Similarly, 553 DEGs were identified based on the stromal score: 385 upregulated and 168 downregulated (Figures 3B–D). The results of the Venn intersection showed that 261 upregulated genes represent the high-score group of the immune and stromal components, and 126 downregulated genes represent the low-score group of the two components. These DEGs (a total of 387 genes) were TME-related genes and determined as indicators of the TME status. The GO enrichment analysis revealed that the TME-related genes almost mapped to the immune-related GO terms, such as neutrophil activation and granulocyte migration (Figure 3E). The KEGG enrichment analysis also mapped to chemokine signaling and B cell receptor signaling pathways (Figure 3F). Therefore, the overall functions of the TME-related genes seemed to map to immune-related activities, indicating that the immune factors are predominant in the TME in glioma.

### Interaction Analysis Post PPI Network and Uni-Cox Regression Analyses

**Figure 4:**
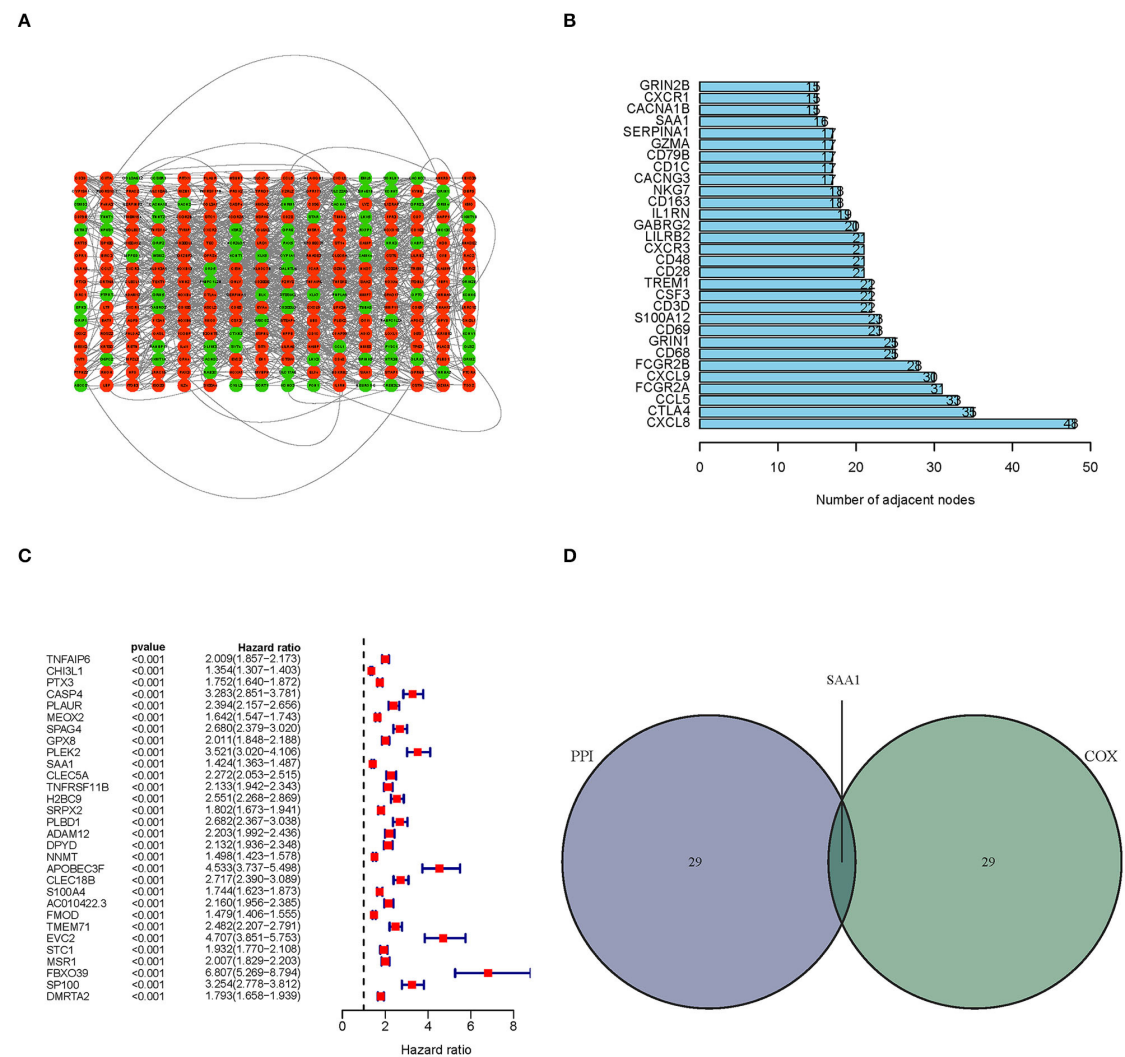
PPI network and univariate Cox regression analyses. **(A)** PPI network was constructed with the TME-related genes using Cytoscape software; the interaction score was > 0.4. Red circles represent the upregulated genes; green circles represent the downregulated genes. **(B)** The map of the top 30 genes in the PPI network is ordered by the number of adjacent nodes. **(C)** Univariate Cox regression with 452 TME-related genes, listing the top 30 factors with *p* < 0.001. **(D)** The intersection of the results from the PPI and Cox regression analyses, SAA1 was identified as a TME-related gene.

Next, we constructed a PPI network based on the STRING database using Cytoscape software. The interactions between 387 TME-related genes are shown in Figure 4A, and the top 30 genes, ranked by the number of nodes, are shown in Figure 4B. Then, a Uni-Cox regression analysis was conducted to assess the survival of glioma patients and determine the significant factors among the 387 TME-related genes. The forest plot of the top 30 factors was shown in Figure 4C. Subsequently, the intersection analysis between the top 30 nodes of the PPI network and the top 30 genes obtained from the uni-Cox regression analysis identified SAA1 as the overlapping gene (Figure 4D).

### Correlation of SAA1 Expression With the Clinical Characteristics and Survival

**Figure 5:**
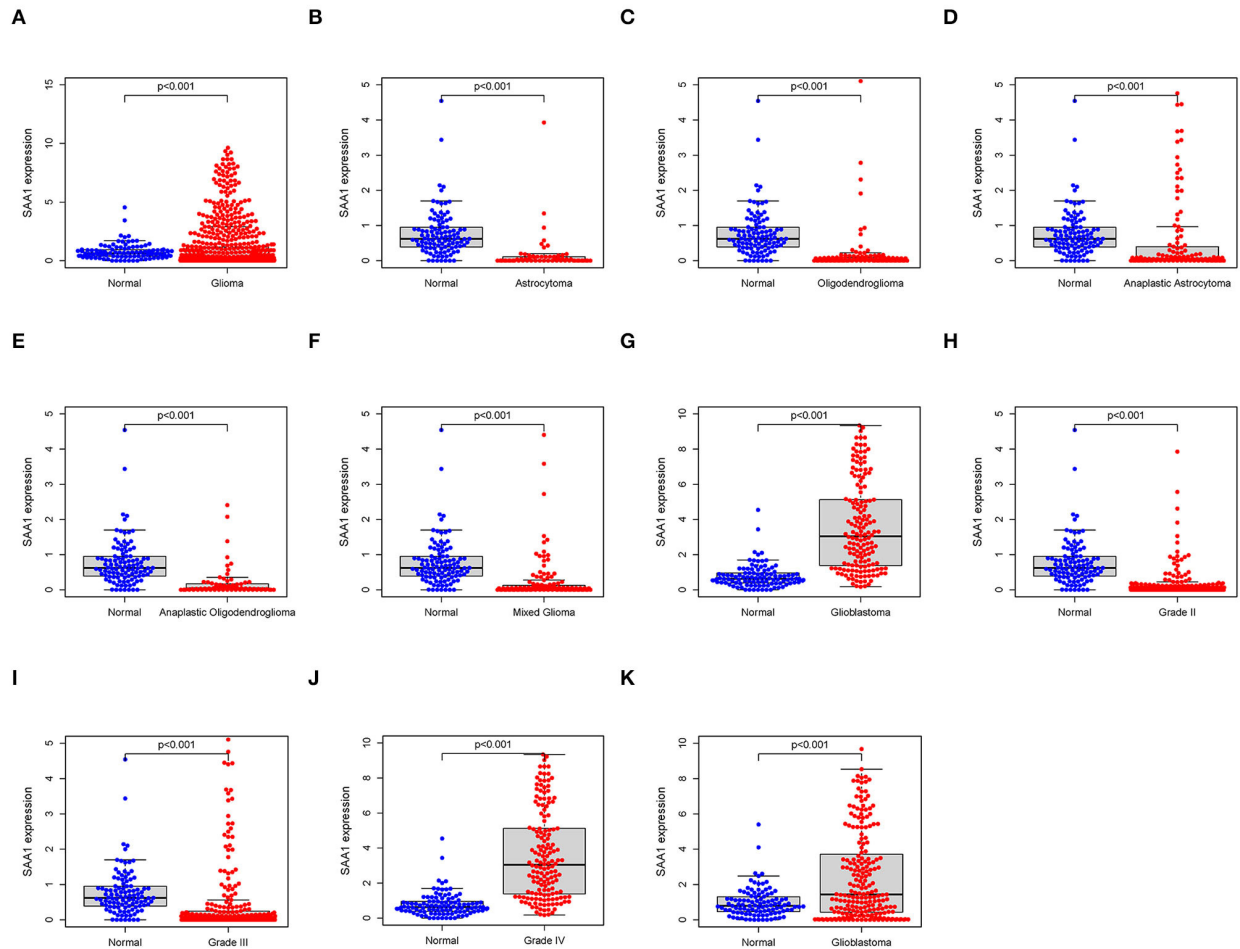
Differential expression of SAA1 between normal and clinical characteristics. **(A)** Differential expression of SAA1 between normal and glioma samples. **(B–G)** Differential expression of SAA1 between normal and different subtypes of gliomas. **(H–J)** Differential expression of SAA1 between normal and different grade gliomas. **(K)** External validation of the differential expression of SAA1 between normal and GBM in the CGGA database.

All the glioma samples were grouped into high- or low-expression groups after comparing the median SAA1 expression levels. SAA1 expression was also evaluated in combination with clinical characteristics. The results are shown in Figures 5A–J. Interestingly, SAA1 expression was significantly lower in normal samples, except in GBM and grade 4 gliomas.

**Figure 6:**
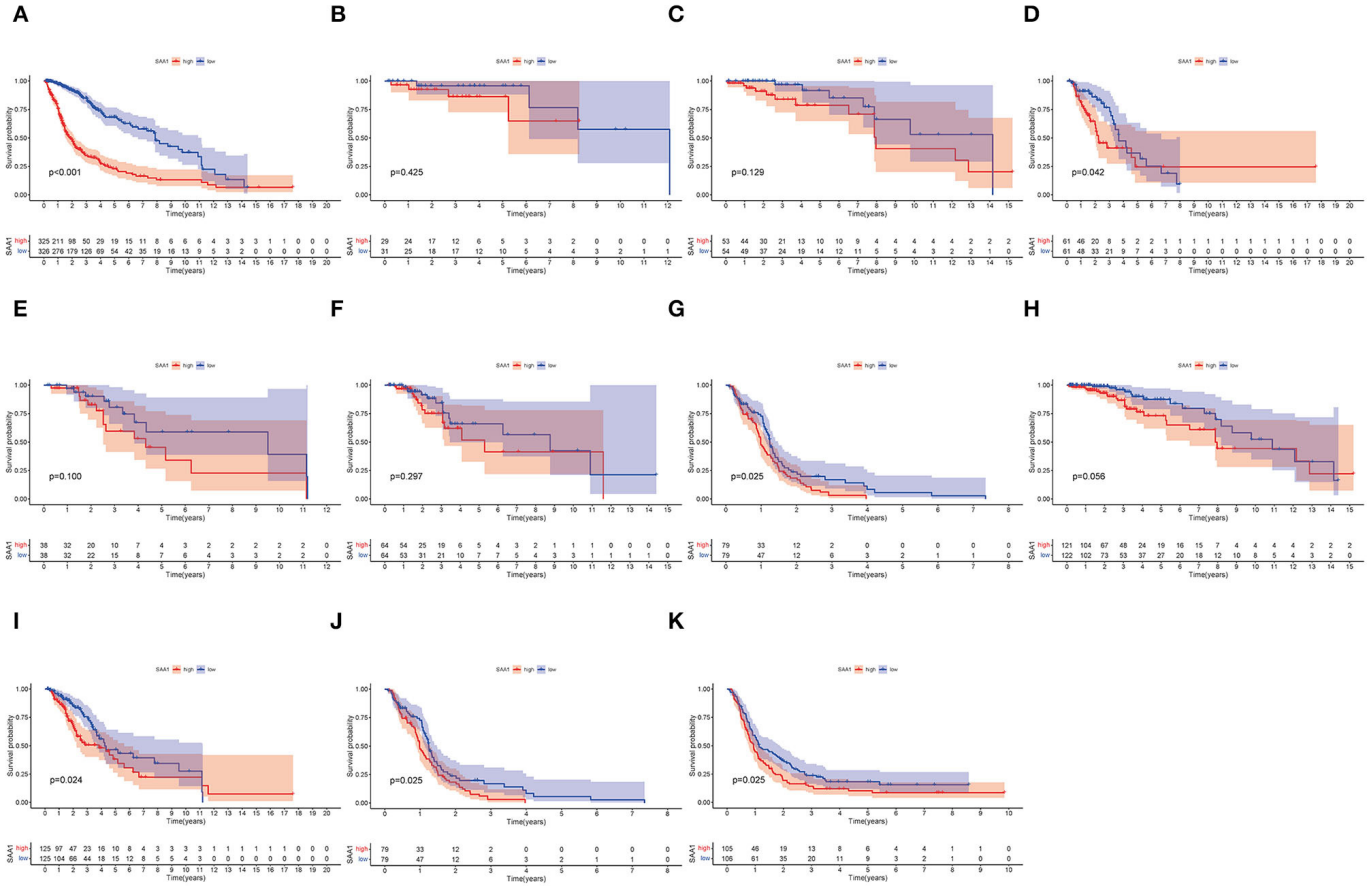
Survival analysis. **(A)** Survival analysis of all glioma patients with different SAA1 expression levels. Patients were grouped into high and low SAA1 expression groups, and the medians were compared, *p* < 0.001. **(B–G)** Survival analysis with different SAA1 expression levels in astrocytoma (A), oligodendroglioma (O), anaplastic astrocytoma (AA), anaplastic oligodendroglioma (AO), mixed glioma (MG), and glioblastoma (GBM), respectively. **(H–J)** Survival analysis in grade 2, 3, and 4 gliomas, respectively. **(K)** External validation in CGGA database and survival analysis in SAA1 high- and low-expression groups for GBM.

Furthermore, the survival analysis (Figure 6) showed that in all glioma patients, low SAA1 expression had prolonged survival compared to high expression (Figure 6A). However, combined with clinical characteristics, we found that in different subtype gliomas (Figures 6B–G), anaplastic astrocytoma (AA), and GBM, SAA1 expression is correlated with survival. Grades 3 and 4 gliomas were statistically significant (Figures 6H–J). The above results indicated that SAA1 expression in the TME was negatively correlated with the prognosis of GBM. Specifically, SAA1 differed between GBM and other gliomas, indicating that SAA1 was the representative TME-related gene of GBM compared to other subtype gliomas.

The external validation in the CGGA database also showed similar results for SAA1 expression level and survival status (Figures 5K, 6K, respectively).

### Construction of a Nomogram and Assessment of SAA1 as a Prognostic Factor

**Figure 7:**
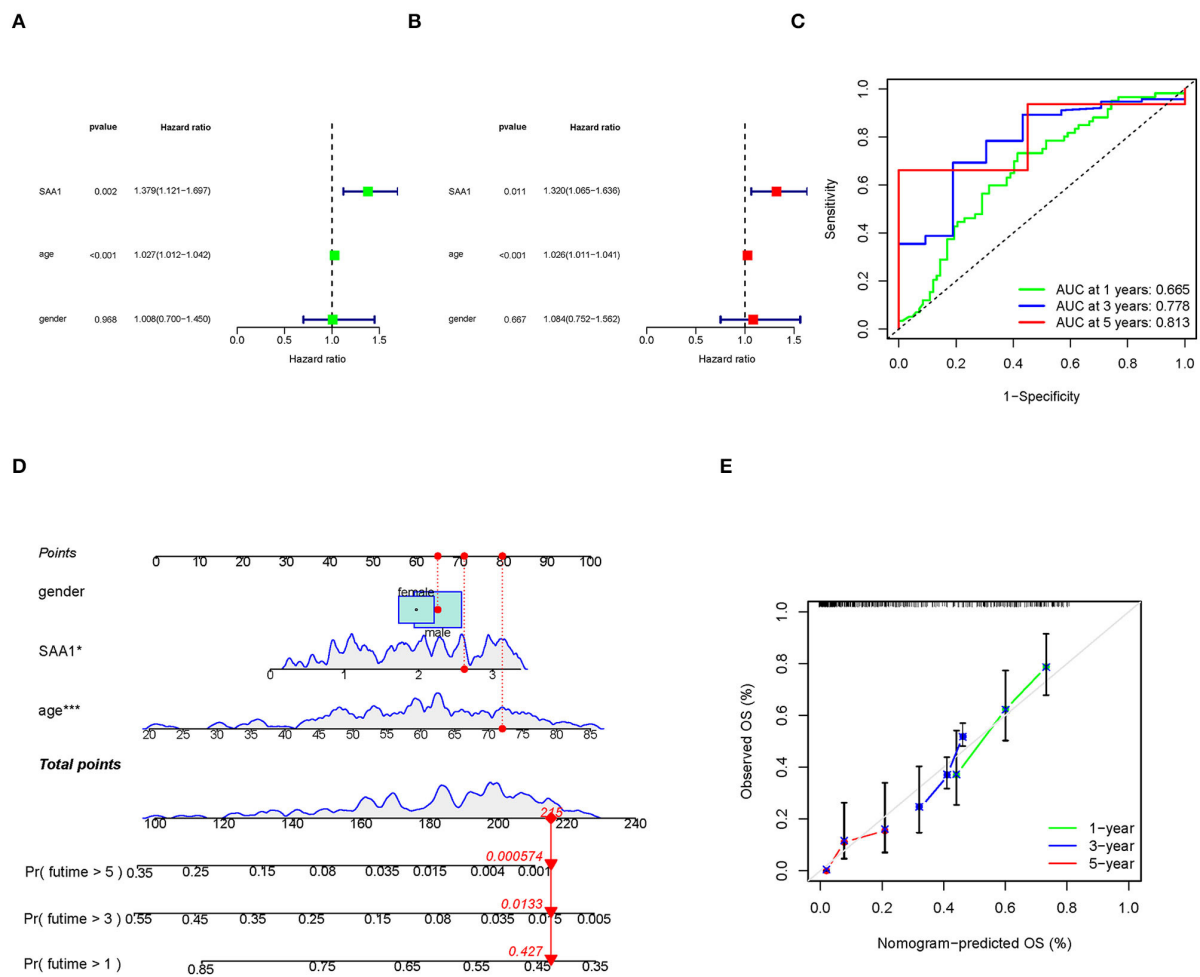
Identification of SAA1 as an independent prognostic factor. **(A)** Uni-cox forest plot of *SAA1, p* = 0.002, HR: 1.379. **(B)** Multi-cox plot of *SAA1, p* = 0.011, HR: 1.320. **(C)** ROC curves of 1-, 3-, and 5-year OS and AUCs are 0.665, 0.778, and 0.813, respectively. **(D)** A nomogram that integrated *SAA1*, age, and gender and predicted the probability of the 1-, 3-, and 5-year OS. **(E)** Calibration curves for 1-, 3-, and 5-year OS, respectively.

To identify SAA1 as an independent prognostic factor, we combined the clinical characteristics, survival time, and survival status of uni-Cox and multi-Cox analyses (Figures 7A,B). In uni-Cox analysis, the hazard ratio (HR) was 1.379, and the 95% confidence interval (CI) was 1.121–1.697 (*p* = 0.002), while in multi-Cox analysis, the HR was 1.320, and the 95% CI was 1.065–1.636 (*p* = 0.011). These values indicated that SAA1 and age were independent prognostic factors for GBM patients, while gender was not a prognostic factor.

To identify the accuracy of the independent factors, we plotted a time-dependent ROC curve to evaluate the sensitivity and specificity of SAA1 on prognosis. As shown in Figure 7C, we also illustrated the outcomes of ROC with the area under the curve (AUC). The 1-, 3-, and 5-year AUC was 0.665, 0.778, and 0.813, respectively.

According to the above results, we established a nomogram for predicting the GBM patients' 1-, 3-, and 5-year OS incidence (Figure 7D). The 1-, 3-, and 5-year calibration plots corroborated the nomogram data with the predicted 1-, 3-, and 5-year OS (Figure 7E).

### WB Analyses

**Figure 8:**
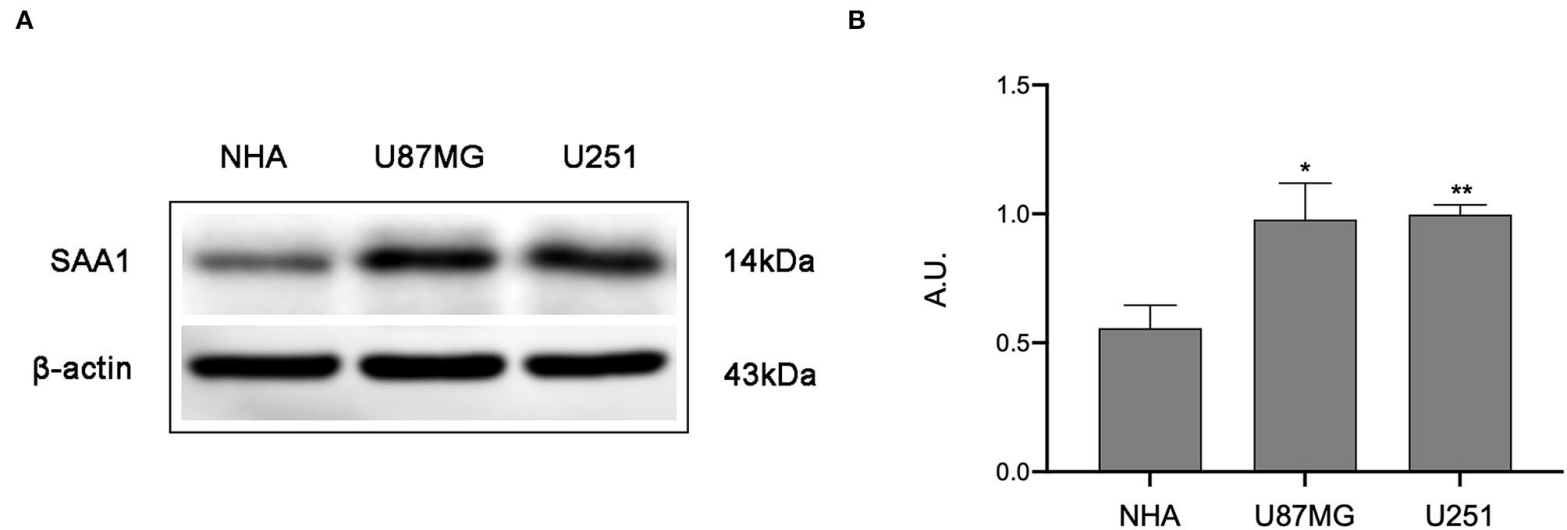
Results of Western blot analysis. **(A)** Western blot was used to detect the expression levels of SAA1 in glioma cell lines (U87MG and U251) and normal noncancerous glial cell lines (NHA). **(B)** Quantification of protein expression corresponding to the Western blot. Data are presented as the mean ± SEM. The A.U. of NHA, U87MG, and U251 were 0.557, 0.978, and 0.997, respectively. ** Represented *p* < 0.01 and * represented *p* < 0.05.

To further confirm the results of bioinformatics analysis, the protein levels of SAA1 were examined in glioma cell lines. As shown in Figures 8A,B, the protein level of SAA1 in glioma cell lines U87MG and U251were significantly upregulated compared to normal glial cells NHA. The Absorbance Unit (A.U.) of NHA, U87MG, and U251 were 0.557, 0.978, and 0.997, respectively.

### SAA1 Is a Potential Indicator of TME Modulation

**Figure 9:**
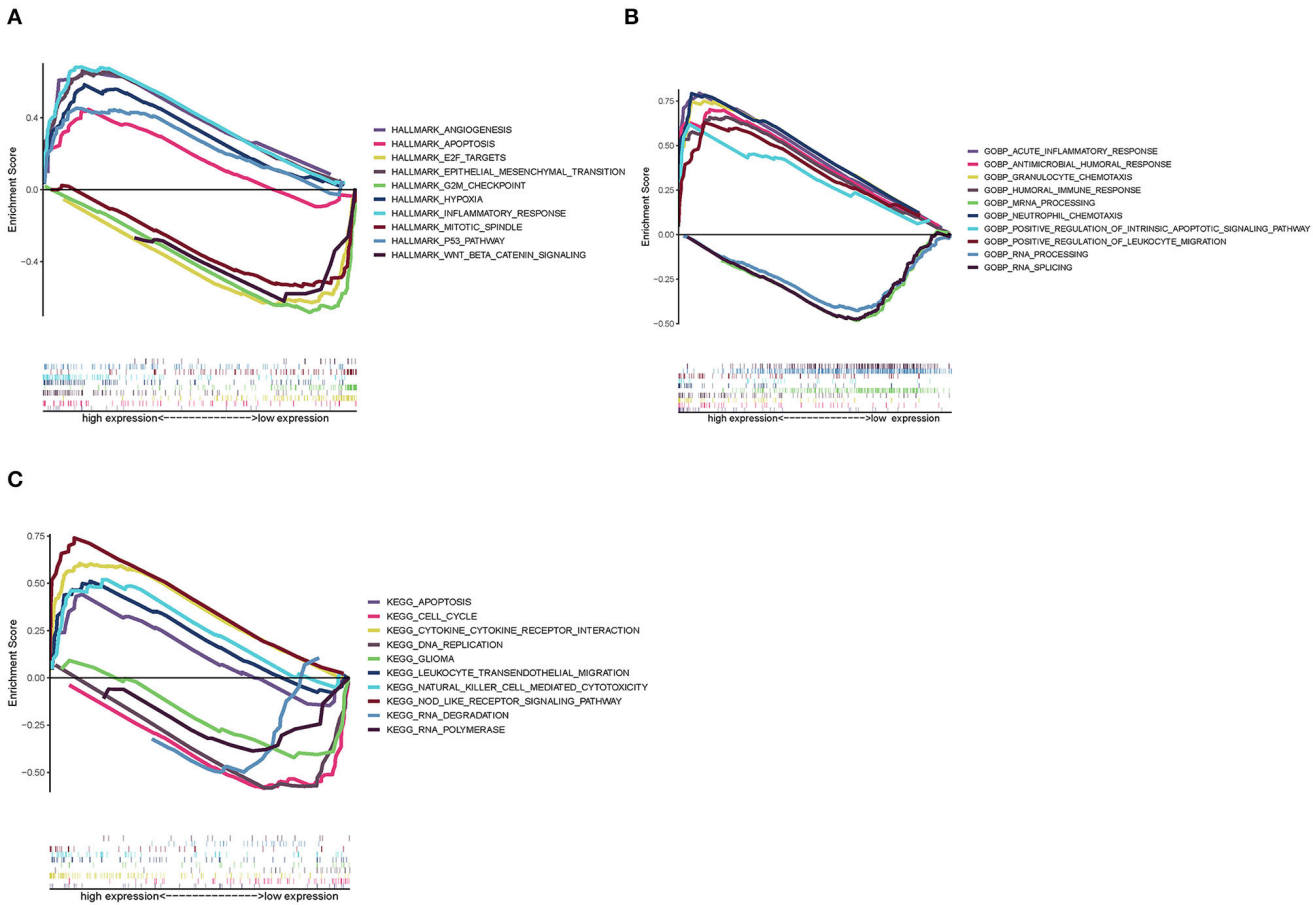
GSEA enrichment analysis for samples with high expression and low expression of SAA1. **(A)** GSEA results in a Hallmark collection. **(B)** GSEA results in GO collection. **(C)** GSEA results in KEGG collection.

Since SAA1 levels are negatively correlated with the survival rate and positively expressed in GBM, Hallmark, GO, and KEGG GSEA analyses were conducted in GBM samples. As shown in Figure 9A, the SAA1 high-expression Hallmark group showed enrichment in angiogenesis and inflammatory response. Conversely, the SAA1 low-expression Hallmark group mainly showed enrichment of genes in the cell cycle control. Figure 9B showed the results of GO GSEA; SAA1 high-expression group was enriched in an acute inflammatory response and antimicrobial humoral response, while the low-expression group was enriched in RNA processing and RNA splicing. Figure 9C shows the KEGG GSEA results, wherein the SAA1 high-expression group was enriched in immune activities, such as cytokine-cytokine receptor interaction, apoptosis, and leukocyte transendothelial migration, while the low-expression group was enriched in DNA replication and cell cycle. These results showed that SAA1 was highly expressed in the physiological process of GBM samples, including tumor progression and immuno-inflammatory responses. Thus, SAA1 might be a potential indicator for the TME status of GBM.

### Correlation of SAA1 With the Proportion of TICs

**Figure 10:**
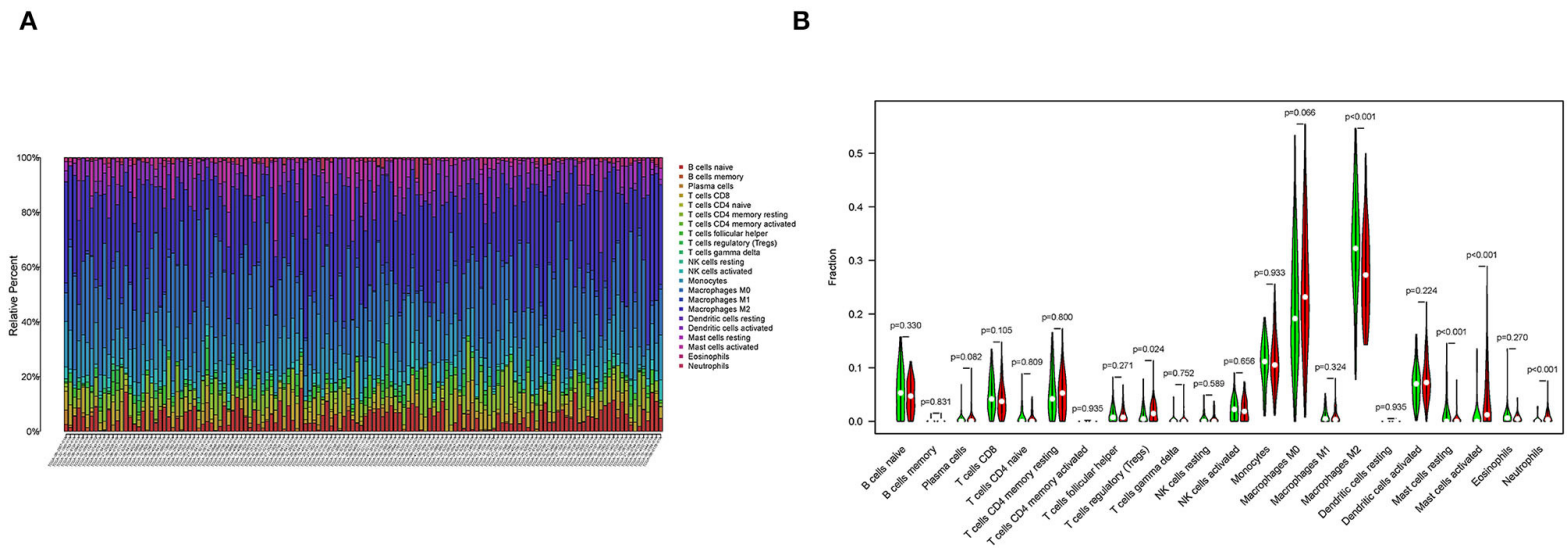
TICs profile of GBM and correlation analysis. **(A)** The bar plot shows the proportion of 22 types of immune cells in GBM samples, and column names are sample IDs. **(B)** The Violin plot shows the differentiation ratio of 22 types of immune cells in GBM samples with low or high expression relative to the median SAA1 expression level. Wilcoxon rank-sum test was used to determine the significance.

To further confirm the correlation between SAA1 expression and the immune microenvironment, the proportion of tumor-infiltrating immune subsets was analyzed using the CIBERSORT algorithm, and 22 types of immune cell profiles were constructed for GBM samples (Figure 10A). The difference analysis revealed that five types of TICs were correlated with SAA1 expression (Figure 10B), including M2 macrophages, neutrophils, activated mast cells, resting mast cells, and regulatory T cells (Tregs). These results provided evidence that SAA1 expression affects the immune activity of the TME.

### SAA1-Associated Immune Checkpoint Genes, TMB, and Drug Sensitivity

**Figure 11:**
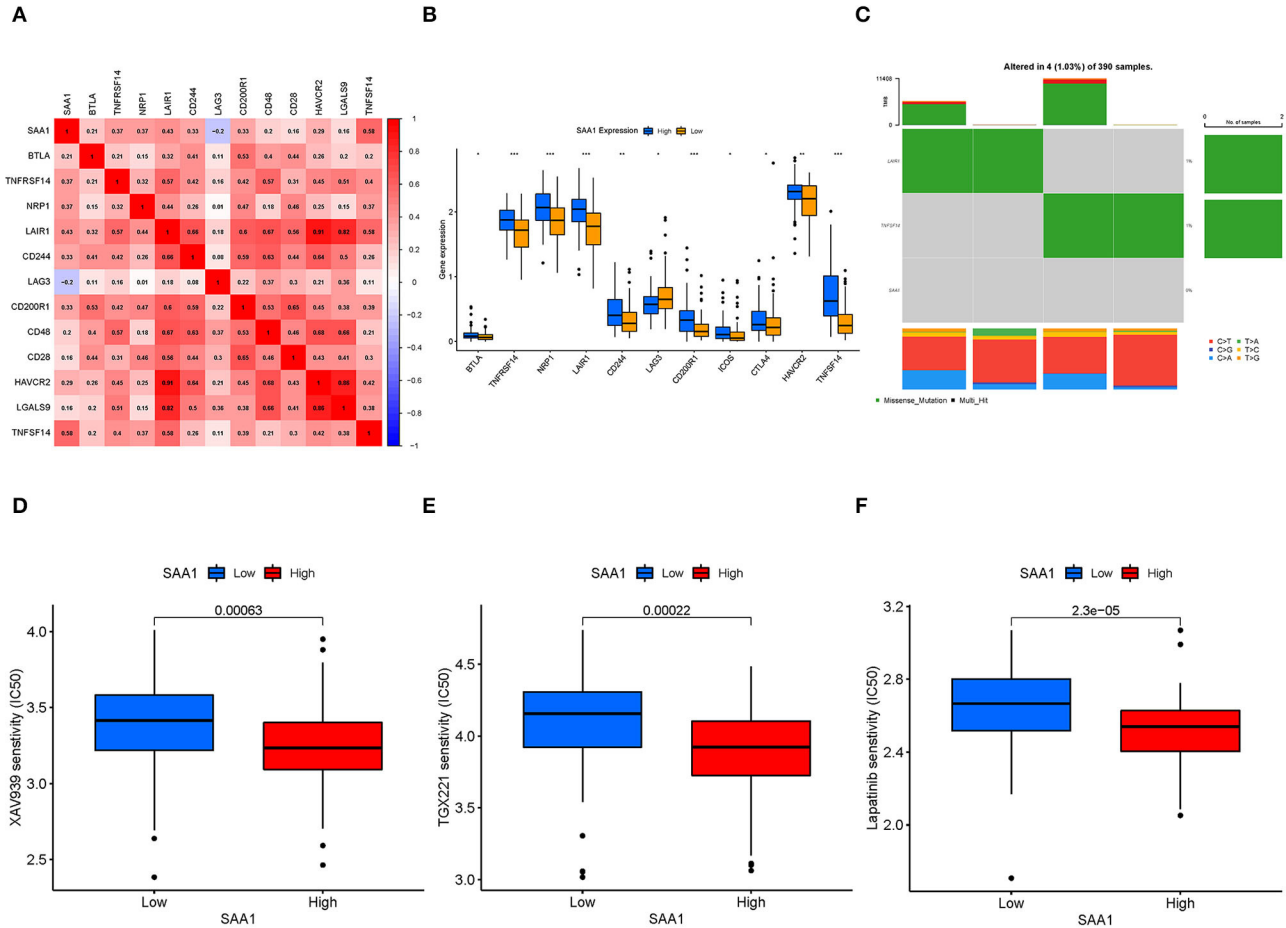
Immune checkpoint genes, TMB, and drug sensitivity. **(A)** Heatmap shows the correlation between SAA1 and immune checkpoint genes, and the numerical value in each box indicates Pearson's correlation coefficient between the two genes. **(B)** Box plot of differentially expressed immune checkpoint genes between SAA1 high- and low-expression groups. **(C)** Heatmap of TMB for SAA1, LAIR1, and TNFSF14 in GBM. **(D–F)** Drug sensitivity of XAV939, TGX221, and lapatinib showed lower IC50 in the SAA1 high-expression GBM group, *p* < 0.001.

Pearson's test was used to calculate the correlation between SAA1 and immune checkpoint genes (Figure 11A); Pearson's correlation coefficient > 0.4 was considered statistically significant. Then, we also identified the differentially expressed immune checkpoint genes between SAA1 high- and low-expression groups, as shown in Figure 11B. The correlation and differential expression analyses identified the following SAA1-associated immune checkpoint genes: LAIR1 and TNFSF14. The TMB results are summarized in Figure 11C. The mutation frequency of SAA1 was 0%, and that for LAIR1 and TNFSF14 was 1%, which indicated almost no mutation of the genes in GBM. Furthermore, the drug sensitivity analyses (Figures 11D–F) revealed that XAV939, TGX221, and lapatinib had a low IC50 in SAA1 high-expression group than in the low-expression group (*p* < 0.001).

## Discussion

Glioma is the most common malignant brain tumor with various subtypes and poor prognosis. In this study, we collected almost all subtypes of glioma samples from TCGA, encompassing astrocytoma (A), oligodendroglioma (O), anaplastic astrocytoma (AA), anaplastic oligodendroglioma (AO), mixed glioma (MG), and GBM. Although the previous treatment could prolong the patients' lives, treatment of gliomas, especially GBM, failed frequently. The oncogenesis and evolution of gliomas are not yet well-understood. Moreover, the complex classification of gliomas, their intense aggressiveness, drug resistance, and easy recurrence make the treatment challenging (2, 13, 14). Recent studies have shown that in addition to conventional methods, such as surgical resection, radiotherapy, and chemotherapy, many biomarkers, such as IDH mutation and *1p/19q* codeletion, could be helpful for the prognosis of gliomas (15). In this study, we identified SAA1, a new biomarker related to the TME of GBM, for the prognosis of this malignant tumor and an indicator to distinguish between GBM and other gliomas.

The tumor microenvironment has been widely implicated in tumorigenesis because it harbors tumor cells that interact with the surrounding cells through the circulatory and lymphatic systems and affect the development and progression of cancer. In addition, non-malignant cells in the TME play critical roles in all the stages of carcinogenesis by stimulating and facilitating uncontrolled cell proliferation (7). The current study aimed to find the core genes related to TME that may have prognostic value and the potential to become a treatment target in clinical practice. Consequently, we identified SAA1 as a biomarker of glioma TME via integrated bioinformatic analysis and further validated the expression of SAA1 in multiple databases and GBM cell lines. The results demonstrated that SAA1 was highly expressed in GBM tissues and cell lines (U87MG and U251). Some other studies showed a similar expression pattern of SAA1 in glioma samples. Knebel et al. found that SAA1 mRNA was considerably higher in GBM than in AGI-III and NN samples. An immunohistochemistry assay revealed cytoplasmic positivity for SAA1 in GBM (16). Furthermore, our study implicated that in GSEA, low-SAA1 expressed group was mainly enriched with mitosis and cell cycle, while the high-SAA1 group predominantly effectuated the immune activity. These results indicated that SAA1 may be an independent target for GBM and has the potential to be an immune therapy target for GBM.

SAA1 is an acute-phase, high-density lipoprotein secreted by the liver in response to infection and tissue injury; thus, its plasma levels are elevated following injury, inflammation, brain trauma, and cancer (17–22). As an essential member of the SAA family, SAA1 has been reported as a risk and prognosis biomarker in tumorigenesis, metastasis, and therapy and is highly expressed in gastric cancer, lung cancer, prostate cancer, endometrial cancer, esophagus cancer, and melanoma, which presented poor prognosis in patients (23–28). Recent studies in glioma also proved that SAA1 knockdown inhibits the phosphorylation of serine/threonine protein kinase B (AKT), which regulates the production of apoptosis-related proteins, such as Bcl2 and Bax, resulting in GBM cell death (29). Furthermore, SAA1 promotes α_V_β_3_-mediated cell migration and invasion in GBM and activates the Erk signaling pathway (30). In this study, bioinformatics analysis further revealed that SAA1 is associated with the TME status, expressed at higher levels in GBM, and is negatively correlated with the survival time. Further analyses demonstrated that compared to different subtype gliomas, SAA1 expression was higher in normal samples only in GBM of grade 4 glioma. In LGGs, SAA1 showed a lower expression than in normal samples. These results indicated that SAA1 might be a distinctive signature for GBM and LGG. In this study, we identified SAA1 as an independent TME-related prognostic marker.

The tumor microenvironment scores are associated with immune cell infiltration, and high TME scores are correlated with a high number of immune infiltrating cells and poor survival of glioma patients (31). In the current study, the TME scores are altered with changing grades and subtypes of gliomas. Moreover, the results of TME scores showed that GBM of grade 4 glioma had the highest scores, which corresponded to a poor prognosis. Furthermore, the TME scores of GBM were > 0, while other grades and subtypes were <0, indicating that GBM had an abundance of immune infiltrating cells, which modified the immune therapy.

The immune cells in TME play a vital role. Herein, we identified five types of TICs, including M2 macrophages, neutrophils, activated mast cells, resting mast cells, and Tregs. These cells infiltrate the tissue surrounding the tumor cells and influence tumor progression and treatment. Tumor-associated macrophages (TAMs) constitute the majority of immune cells in brain tumors, accounting for up to 30% of the tumor mass (32). Some studies have demonstrated that large numbers of infiltrating TAMs are closely associated with poor prognosis (33), and the “M2” phenotype promotes tumor progression via secretion of immunosuppressive cytokines and factors promoting angiogenesis (10, 33). M2 macrophages have been identified to exert a modulatory role in glioma progression (34). The most abundant granulocyte in humans is the neutrophil, constituting up to 70% of the total leukocyte population in the body (35). In this study, neutrophils were positively correlated with SAA1 expression. Contrary to their pro-inflammatory function during infections, neutrophils have been frequently reported to promote tumor progression and metastasis in recent years (36). However, experimental validation regarding the correlation between SAA1 expression in tumor tissues and immune cell infiltration is lacking, which is one of the major limitations of this study.

The treatment failure of gliomas is a common occurrence, promoting the development of new technologies and interest in immunotherapy (immune checkpoint molecule, TAM, dendritic cell vaccine, CAR-T, TME, and a combination of several efficacious methods) (37). Herein, we explored the correlation of SAA1 with immune checkpoint genes and drug sensitivity. TME has been associated with drug resistance and immune suppression. In lung cancer, LAIR1 is upregulated, induces T cell exhaustion, and abrogates resistance to anti-PD-L1 (38). TNFSF14-mediated vascular remodeling activates the endothelia and induces intra-tumoral high endothelial venules, which are specialized blood vessels for lymphocyte infiltration (39). In this study, we found that SAA1 is positively related to the expression of LAIR1 and TNFSF14. Previous research has postulated a convergence between high expression of LAIR1 and TNFSF14 and the immunosuppressive microenvironment in GBM (40, 41). TNFSF14 has also been linked to an imbalance in adaptive immune resistance pathway gene expression, which may affect GBM prognosis (40). These findings support our hypothesis that SAA1 is a novel TME-related gene for GBM and its value as an immunotherapeutic target.

XAV939 is a small molecule inhibitor of the Wnt-signaling pathway that blocks Wnt-signaling in cancer cell lines, resulting in a dramatic stabilization of the axin protein and inhibiting the β-catenin-regulated transcription (42). TGX221 inhibits proliferation and induces apoptosis in glioblastoma cells (43). Lapatinib is an EGFR inhibitor. A pilot phase II study showed that pulse high-dose lapatinib in addition to standard therapy is a tolerable and safe regimen for newly-diagnosed GBM (44). Drug sensitivity analyses identified three drugs with low IC50 in SAA1 high-expression group, confirming that SAA1 high expression in GBM is an indicator of drug sensitivity.

Taken together, the current study described the correlation of SAA1 with the GBM microenvironment. Immune therapy, WB, and bioinformatics identified SAA1 as an independent marker. However, the correlation between SAA1 and immune checkpoint genes and immune drugs needs to be explored further.

## Conclusion

SAA1 may be a distinguishing factor between GBM and other glioma subtypes and a new biomarker for determining the TME status and patients' survival. The principal role of SAA1 shifts from cell cycle and mitosis to immune activity with changing expression levels. SAA1 is a drug sensitivity indicator for XAV939, TGX-221, and lapatinib in GBM immune treatment because it upregulates the expression of LAIR1 and TNFSF14.

## Data Availability Statement

The original contributions presented in the study are included in the article/supplementary materials, further inquiries can be directed to the corresponding author/s.

## Author Contributions

YC and ZN: conceptualization and funding acquisition. KC: methodology, software, resources, writing—original draft preparation, and visualization. KC and XJ: validation, formal analysis, and investigation. KC and BW: data curation. ZN and YC: writing—review and editing. YC: supervision. KC and YC: project administration. All authors have read and agreed to the published version of the manuscript.

## Funding

This research was partially funded by Jilin Province Science and Technology Department, grant numbers 20200201510JC and 20210402021GH and the Education Department of Jilin Province, grant number JJKH20211147KJ.

## Conflict of Interest

The authors declare that the research was conducted in the absence of any commercial or financial relationships that could be construed as a potential conflict of interest.

## Publisher's Note

All claims expressed in this article are solely those of the authors and do not necessarily represent those of their affiliated organizations, or those of the publisher, the editors and the reviewers. Any product that may be evaluated in this article, or claim that may be made by its manufacturer, is not guaranteed or endorsed by the publisher.
